# A Rare Case of a Neuroendocrine Tumor at the Duodenum

**DOI:** 10.7759/cureus.57696

**Published:** 2024-04-06

**Authors:** Murad Qirem, Shahd Yaghi, Byron Okwesili, Raed Atiyat, Yatinder Bains

**Affiliations:** 1 Internal Medicine, Saint Michael's Medical Center, Newark, USA; 2 Gastroenterology and Hepatology, Saint Michael's Medical Center, Newark, USA; 3 Gastroenterology, Saint Michael's Medical Center, Newark, USA

**Keywords:** malignant, bulb, duodenum, tumor, neuroendocrine

## Abstract

Neuroendocrine tumors (NETs) are slow-growing cancers derived from neuroendocrine cells that typically affect the pancreas, lungs, and gastrointestinal tract. A rare form can develop in the duodenum and can be difficult to diagnose and treat. The case below describes a rare incidence of a well-differentiated duodenal bulb NET in a 77-year-old man who had early satiety and persistent dyspepsia. Endoscopy, biopsies, and immunohistochemistry staining were used to confirm the diagnosis. According to the features of the tumor, management techniques, including endoscopic, surgical, and medicinal procedures, are being implemented.

## Introduction

Neuroendocrine tumors (NETs) are malignant, slow-growing tumors that arise from neuroendocrine cells. They most commonly occur in the gastrointestinal tract (48%), lung (25%), and pancreas (9%) but may also develop in many other organs, including the breast, prostate, thymus, and skin [1].

Duodenal neuroendocrine tumors (NETs) are a rare subtype of gastroenteropancreatic neuroendocrine tumors (GEP-NETs) developing from neuroendocrine cells in the duodenum, which is the initial part of the small intestine. Even though they are uncommon, duodenal NETs have been increasing in incidence; this could be because of advancements in diagnostic methods and increased provider awareness [2]. These tumors have distinct clinical and pathological characteristics, which frequently make diagnosis and treatment difficult.

In the following case, we report a rare occurrence of duodenal NETs. This specific case stands out as exceptionally unique compared to previously reported cases, primarily due to its uncommon location, which was at the duodenal bulb.

## Case presentation

**Figure 1:**
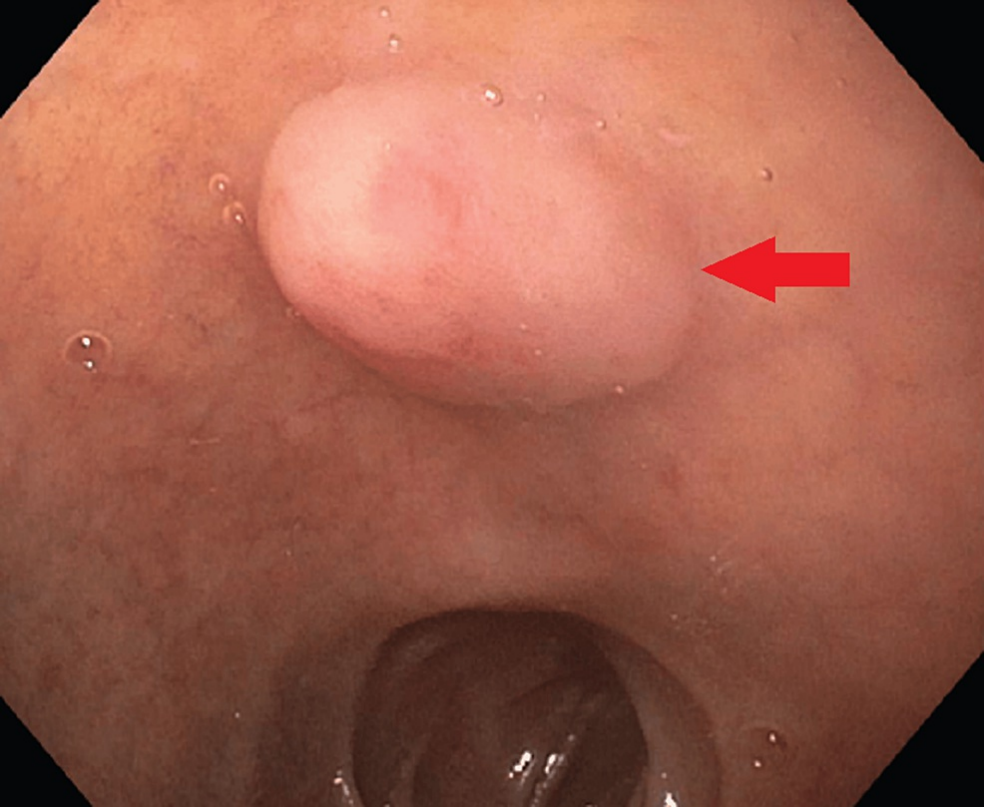
Endoscopic appearance of neuroendocrine tumor. The arrow above points at the mass in the duodenum.

**Figure 2:**
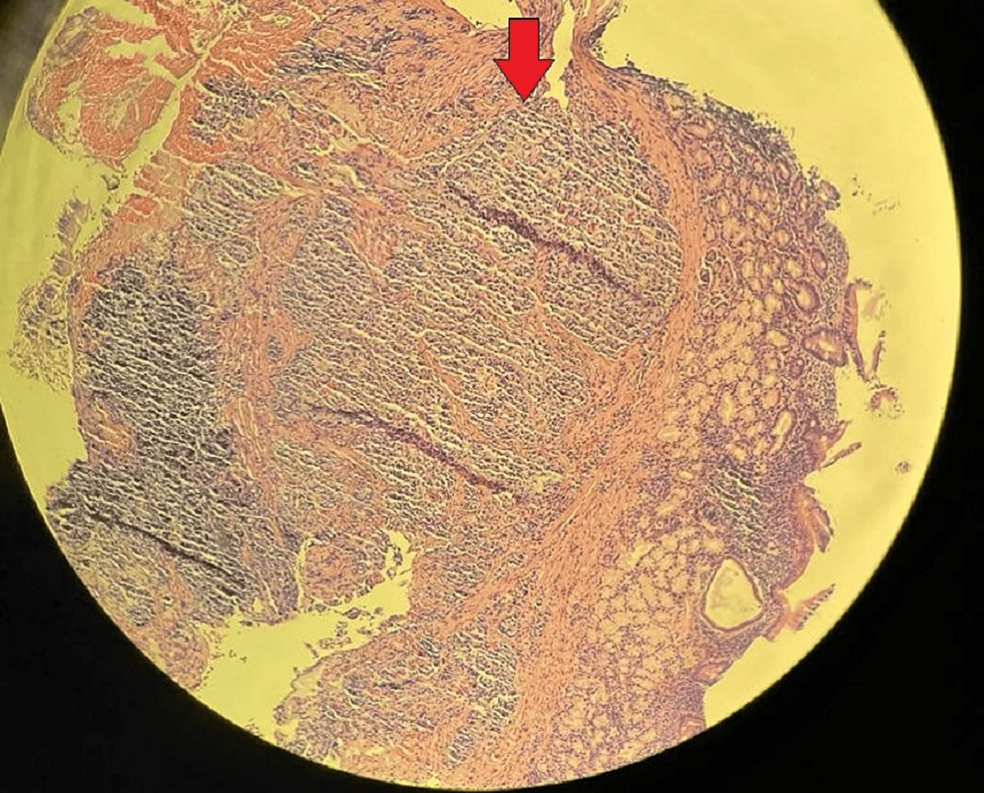
Histology of the mass. Neuroendocrine tumor cells.

This is the case of a 77-year-old man who reported dyspepsia and early satiety when he visited the clinic. Symptoms were persistent for six months before presentation, and they were unresponsive to medications such as proton pump inhibitors. An endoscopy was conducted due to his symptoms (Figure 1), and the results showed that he had a duodenal bulb mass. A well-differentiated (grade 1) neuroendocrine tumor was confirmed by a biopsy of the mass (Figure 2). The neuroendocrine origin of the tumor was demonstrated by positive results for synaptophysin, chromogranin A, and S100 in subsequent immunohistochemical investigations. Ki-67 staining was used to measure the proliferative index, which came out to be low at 2%, suggesting a slow rate of cell proliferation. Furthermore, it was found that the mitotic rate was low, ranging from 0 to 1 mitotic figure per two square millimeters.

Based on these findings, we will schedule the patient for an endoscopic ultrasound (EUS) to accurately stage the tumor. Following staging, he will undergo an additional outpatient workup to determine the most appropriate treatment plan, which may include a combination of endoscopic, surgical, and medical interventions tailored to his specific condition.

## Discussion

Duodenal NETs account for a small proportion of all NETs, with a reported incidence rate of approximately one to two cases per 100,000 individuals. While they can occur at any age, duodenal NETs are commonly diagnosed in individuals between the ages of 40 and 60 years [3].

The clinical presentation of duodenal NETs varies widely and can range from asymptomatic incidental findings to symptomatic cases with gastrointestinal bleeding, abdominal pain, or even obstructive jaundice if it blocks the ampulla of Vater [4]. The nonspecific nature of symptoms often leads to delayed diagnosis, making it crucial for healthcare providers to maintain a high index of suspicion, especially in patients with recurrent abdominal complaints.

Accurate diagnosis of duodenal NETs involves a combination of imaging studies, endoscopic procedures, histopathological analysis, and immunohistochemical staining. Endoscopic ultrasound (EUS) and multiphasic computed tomography (CT) scans are valuable tools for visualizing the tumor, determining its size, and assessing its local invasion and metastasis. Histological examination, including immunohistochemistry, is essential for confirming the diagnosis and characterizing the tumor's grade and stage [4]. Immunohistochemical stains are essential in identifying neuroendocrine differentiation within tumor cells and distinguishing NETs from other malignancies with similar histological features. Commonly used markers include chromogranin A and synaptophysin, which are highly sensitive for detecting neuroendocrine differentiation. Additionally, Ki-67, a marker of proliferation, is crucial in determining the tumor grade, helping to stratify NETs into well-differentiated (low-grade) and poorly differentiated (high-grade) categories. The combination of these immunohistochemical stains provides valuable information to pathologists, aiding in accurate diagnosis, grading, and guiding appropriate treatment strategies for patients with NETs [5].

The management of duodenal NETs requires a multidisciplinary approach, with treatment strategies tailored to the tumor's size, location, grade, and metastatic spread. Surgical resection remains the primary curative option, especially for localized tumors. Additionally, advancements in targeted therapies, such as somatostatin analogs (e.g., lanreotide), have shown promising results in controlling tumor growth and managing symptoms in patients with unresectable or metastatic disease [6].

## Conclusions

In conclusion, duodenal NETs pose diagnostic and therapeutic challenges due to their rarity and varied clinical presentation. However, ongoing research and advancements in diagnostic techniques and targeted therapies are improving the outcomes and quality of life for patients with this rare malignancy.
